# Employee ambidextrous job crafting in organizations: theoretical model, scale development, and dual-path effects mechanisms

**DOI:** 10.3389/fpsyg.2026.1767375

**Published:** 2026-05-01

**Authors:** Yiming Dai, Wenjin Wang, Maria Tims, Xiaoyu Guan, Haibo Yu

**Affiliations:** 1 Chinese Academy of Personnel Science, Beijing, China; 2 Department of Innovative Social Work, City University of Macau, Taipa, Macao SAR, China; 3 Department of Management and Organisation, School of Business and Economics, Vrije Universiteit Amsterdam, Amsterdam, Netherlands; 4 School of Government, Beijing Normal University, Beijing, China

**Keywords:** ambidexterity theory, dual-path effects mechanisms, job crafting, regulatory focus theory, scale development

## Abstract

**Introduction:**

While scholars have devoted considerable attention to integrating regulatory-focus theory into the job crafting field, there is no consensus on the fundamental definition, structure, and relationship between the promotion- and prevention-oriented job crafting.

**Methods:**

In study 1 (N = 923), the ambidextrous job crafting scale is 4 developed and tested for its factor structure and reliability. In Study 2 (N = 387), the dual-path effects mechanisms of the ambidextrous job crafting are examined using a lagged study design.

**Results:**

By integrating ambidexterity theory, we develop and validate a scale to measure ambidextrous job crafting behavior using two separate studies conducted in the Chinese context. Ambidextrous job crafting is defined as the employees’ behavior strategies of combining promotion- and prevention-oriented job crafting. The results indicate that there are two distinct forms of job crafting (promotion-oriented and prevention-oriented) and five dimensions for each form (cognitive, emotion, skill, task, and relationship). These dimensions could be measured with 41 items with satisfying reliability and validity. Results of structural equation and bootstrap models indicate that promotion-oriented job crafting is negatively correlated with turnover intention through work meaning, while prevention-oriented job crafting is positively correlated with turnover intention through work meaning and emotional exhaustion. Additionally, the difference and interaction between promotion- and prevention-oriented job crafting are negatively correlated with turnover intention through work meaning and emotional exhaustion.

**Discussion:**

Overall, our results underscore the importance of treating job crafting as a dual-path, multi-dimensional concept. The unique insights provide an essential aspects of job crafting.

## Introduction

Today’s workplace is characterized by a higher level of uncertainty and complexity, making flexibility and initiative of employees increasingly appealing for both employees and researchers of work design theory. Defined as the proactive behaviors to maintain or improve work design (Tims and Bakker, 2010; Wrzesniewski and Dutton, 2001), job crafting has been examined using various frameworks, among which the regulatory focus theory provides a prominent perspective. By theoretically accounting for beneficial and detrimental job crafting effects, regulatory focus theory is proposed to explain “how” employees’ self-initiate changes to their jobs via promotion or prevention focus (Lichtenthaler and Fischbach, 2019). Promotion-oriented job crafting represents a “gains” approach whereby the employee adds to and extends existing job aspects to make sure “gains” occur and to avoid “non-gains”; while prevention-oriented job crafting represents active changes to one’s job that will prevent negative outcomes.

Although previous studies have confirmed promotion- and prevention-oriented job crafting (Bindl et al., 2019), their interaction remains understudied (Mäkikangas, 2018). While prevention-oriented job crafting as a protective health factor may protect individuals’ resources (Bruning and Campion, 2018) and avoids negative outcomes (Tims et al., 2012), empirical evidence has been inconsistent (Lichtenthaler and Fischbach, 2019). According to regulation-focus theory, promotion focus and prevention focus were conceptualized as two independent dimensions rather than opposite ends of a single spectrum (Higgins, 1997, 1998), suggesting that both dimensions can be observed simultaneously in a single employee. However, many existing studies treated them as separate or unrelated traits (Gamache et al., 2015), lacking an coherent and integrative framework to capture their dynamics.

Besides, the role of emotional crafting has long been understudied in job crafting literature (Tian, 2022), despite growing recognition of the importance of employees’ emotional issues during the Industrial 4.0 era, especially in the context of enhancing employees’ adaptability to AI (Teng et al., 2024; Sha et al., 2025). Previous research fails to systematically examine the process and mechanisms of emotional crafting as an independent form; corresponding measurement tools are thus limited and in need of further development.

To address these gaps, this study introduces an ambidextrous perspective into job crafting research, proposing that the promotion- and prevention-oriented job crafting are dynamically interconnected, and thus can be understood within a unified framework. We also extend the framework by incorporating an emotional dimension and developing a set of corresponding measurement tools. A series of studies are presented in the following sections to conceptualize, measure, and examine ambidextrous job crafting. Study 1 develops and validates an ambidextrous crafting scale and examines its psychometric properties, including scale reliability and factorial validity. Study 2 tests a dual-process model to further examine the effects of ambidextrous job crafting on different work outcomes and the mechanisms through which negative effects of prevention-oriented job crafting may be alleviated.

## Theoretical background

### Ambidexterity in the job crafting process

Scholars of work design and motivation have been debating the incorporation of “ambidexterity” into the job design field (Parker, 2014). Ambidexterity literally means the ability to use both hands with equal ease (Rosing et al., 2011). For employees, ambidexterity is the extent to which employees pursue both explorative and exploitative activities in their work roles (Kauppila and Tempelaar, 2016). Individual exploitation involves behaviors geared toward the refinement and extension of existing assets, competencies, and knowledge, whereas exploration involves behaviors intended to gain broader knowledge and advance new or alternative opportunities.

Two essential characteristics of job crafting lead us to the argument that job crafting requires ambidexterity. First, the two seemingly opposing forms of job crafting – promotion- and prevention-oriented job crafting – are both connected to exploration and exploitation activities, although through different paths. Second, job crafting is not a unidimensional process but a complex and non-linear cycle of exploration and exploitation tasks.

#### Promotion-oriented job crafting, prevention-oriented job crafting, and exploration-exploitation activities

Regulatory focus theory argues that people utilize two distinct systems for self-regulation: promotion focus and prevention focus (Higgins, 1997, 1998). A promotion focus motivates individuals to frame situations in terms of gains *versus* non-gains. Individuals with a promotion focus approach gains, rewards, and pleasure while avoiding situations that do not involve gains (i.e., non-gains). In contrast, a prevention focus urges individuals to understand situations in terms of losses *versus* non-losses, prompting them to prefer situations without losses (non-losses) and to avoid loss, pain, and punishment.

Although prevention and promotion focuses represent different mentalities, both are connected with exploitation and exploration activities, which are usually connected with separate focuses (Kark and Van Dijk, 2007). Because long-term maximization of gains is dependent on the level of engagement in the exploration activities, employees with a promotion focus have been reported to have a strong tendency to engage in exploration activities that broadens their worldview. For example, promotion-oriented cognitive crafting requires employees to reframe their job as a meaningful entity (Berg et al., 2010). As a result, employees would face their task and colleagues with a more positive and optimistic attitude, become more motivated in getting more resources, and build up confidence to complete challenging tasks. Meanwhile, we argue that prevention focus can also promote participation in exploratory activities to ensure a “non-losses” state. For instance, when an organization is in urgent need of new projects for higher profits, prevention focus employees may explore riskier strategies to lower their boss’s expectations, thus avoiding failing the expectation despite their preference for prevention strategies. In other words, given the proper context, prevention focus may prompt employees to participate in exploratory activities.

Similarly, we posit that promotion- and prevention-oriented job crafting both cover various exploitative tasks. Prevention focus individuals, who typically emphasize obligations, minimal goals, and safety, are more likely to concentrate on exploitation activities characterized by refinement, implementation, and efficiency (Mom et al., 2009). Nevertheless, according to regulatory focus theory, promotion focus employees are just as likely to perform exploitation activities under the influence of factors like situational cues (Friedman and Förster, 2001). For example, if employers set an expectation to minimize financial losses from failed projects, promotion focus employees may adjust their strategic tendency to a more conservative and exploitative approach, focusing on their duties to carefully ensure their “gains.”

#### The flexibility to switch and ambidextrous job crafting

The second key characteristic of job crafting is its inherent complexity. Existing literature revealed that both promotion- and prevention-oriented job crafting are theoretically valuable; however, the symbiotic nature of the two was often ignored (Mäkikangas, 2018). For example, an employee may wish to add an extra task to his job (exploration activities) but faces resistance from a supervisor, who wants him to stay focused on his main job. In such complicated situations, work tasks usually unfold in unpredictable and nonlinear ways rather than neatly and successively. Therefore, employees are expected to proactively engage in a dynamic blend of both prevention and promotion strategies, depending at least partially on situational and task demands.

In the current study, we use the term flexibility to describe the situation in which employees switch deftly between promotion- and prevention-oriented job crafting to capitalize on the synergy of the two. For example, an employee might first gain a supervisor’s trust by fulfilling their primary responsibilities (exploitation activity) before pursuing the additional tasks they intended to work on (exploration activity). In other words, scoring differently on the two strategies over time does not necessarily mean that the employees have changed their focus. It is possible that they are just switching between different strategies in response to new challenges in their jobs (Berg et al., 2010). Interactions among different types of crafting have been documented by previous studies. For instance, different forms of approach crafting can buffer the relationship between avoidance crafting and different outcomes (Tims et al., 2022). We suggest that, methodologically, an employee can be defined as “ambidextrous” in two ways. First, an ambidextrous employee usually scores high on both promotion- and prevention-oriented job crafting, indicating a synergistic effect. Second, given that ambidexterity consists of both exploration and exploitation, it can be enhanced through any factor that increases activity, provided that this increase is not at the expense of decreasing the other (Kauppila and Tempelaar, 2016). An individual’s ambidextrous job crafting is more effective when increased promotion-oriented job crafting is coupled with a sustained high level of prevention-oriented job crafting.

**Figure 1:**
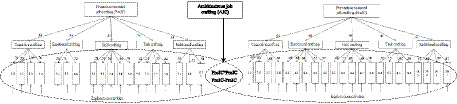
Proposed model of ambidextrous job crafting.

Taken together, we suggest that prevention- and promotion-oriented job crafting, as well as exploration and exploitation activities, should all be integrated into the framework of ambidextrous job crafting together with the relationship between promotion- and prevention-oriented job crafting (Figure 1). This framework suggests a cyclic and interactive relationship between promotion and prevention focuses. Promotion- and prevention-oriented job crafting are complementary and interdependent, each contributing to employees’ careers and the development of their organizations (Mäkikangas, 2018). Ambidextrous-oriented employees tend to combine promotion- and prevention-related job crafting according to the circumstance, switching smoothly between exploration and exploitation activities (Rosing et al., 2011). As an extension to the existing job crafting typology, ambidextrous job crafting is defined from a behavioral perspective as a multifaceted construct instead of a set of independent strategies.

### Toward a comprehensive ambidextrous job crafting model

Like promotion- and prevention-oriented job crafting, ambidextrous job crafting also consists of multiple forms. Building on Wrzesniewski and Dutton’s (2001) seminal work, Bindl et al. (2019) posited that individual needs drive employees to engage in distinct job-crafting strategies - task, relationship, skill, and cognitive crafting - and that work-related regulatory focus is associated with promotion- or prevention-oriented forms of these strategies. Specifically, employees engage in task crafting actively change “the number, scope, or type of job tasks done at work” compared to their prescribed formal job (Wrzesniewski and Dutton, 2001; p. 185). Likewise, employees engage in relationship crafting by changing the way they interact with others at work (Wrzesniewski and Dutton, 2001). Recently, skill crafting is also developed as a necessary type of job crafting, representing employees’ self-initiated efforts to change their skills at work to better carry out their jobs (Wrzesniewski et al., 2012). Moreover, cognitive engagement happens when employees go through a series of internal rather than behavioral changes, leading them to perceive their roles differently (Wrzesniewski and Dutton, 2001).

In addition to the existing forms, we propose that the crafting of emotion should also be incorporated into the ambidextrous model. Emotional crafting has repeatedly appeared in the theoretical and empirical studies of job crafting. For example, in the foundational literature, Wrzesniewski and Dutton has proposed “reflecting on the emotional nature of work situations and calibrating responses appropriately, and relational asking” (2001). In addition, “Use my thoughts to put myself into a good mood at work” and “Use my thoughts to get me out of a bad mood at work” also appeared in the Role–Resource Approach–Avoidance Job Crafting Measure (Bruning and Campion, 2018). However, previous studies have either approached the issue from a rational perspective, treating emotion as secondary to cognition (Jasper, 1998); or has proposed emotional crafting dimensions only at a theoretical dimension but failed to develop corresponding measurement tools (Tian, 2022; Eguchi et al., 2016). In our study, we concept emotional crafting reflects individuals’ self-initiated efforts to change their emotional experience and expression at work. It aligns with the prevention- and promotion-focus framework of regulatory focus theory, which examines how people self-regulate when coping with different situations (Higgins, 1997) that are usually stressful and require the simultaneous consideration of one’s emotions (Zhang et al., 2019). Therefore, we believe that an additional emotional form is needed when discussing job crafting.

*Hypothesis 1*: Ambidextrous job crafting can be structurally divided into two orientations (promotion-oriented and prevention-oriented); each orientation further contains five forms (cognitive, emotional, skill, task, and relationship)

### Relationships between ambidextrous job crafting and work meaning, emotional exhaustion, and turnover intention

According to the job crafting process model (Lazazzara et al., 2020), work meaning and emotional exhaustion are both important outcomes of job crafting. In addition, conservation of resources theory (COR; Hobfoll, 1989) provides an integrated theoretical framework, detailing how job crafting damages or benefits crafters’ well-being in response to resource fluctuation over time (Li et al., 2022).

**Figure 2:**
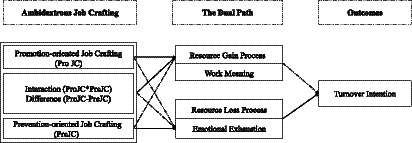
Proposed model of this study.

Based on the COR theory, we believe that emotional exhaustion exemplifies the resource loss path (Whitman et al., 2014), while work meaning represents the resource gain path (Zou et al., 2022). Therefore, we aim to examine whether ambidextrous crafting can affect turnover intention (Wang et al., 2024; Zhang and Li, 2020) through work meaning (source gain process) and emotional exhaustion (resource loss process) (Figure 2).

#### Promotion- and prevention-oriented job crafting and their effects

Work meaning is usually considered an important psychological state (Steger et al., 2012). It is defined as “[t]he degree to which the employee experiences the job as one which is generally meaningful, valuable, and worthwhile (Hackman and Oldham, 1976)”. Emotional exhaustion is a health-related and motivational concept, which can be defined as “[t]he feelings of being emotionally overextended and exhausted by one’s work (Maslach et al., 2001)”. By aligning one’s job to their needs and values or creating intrinsically motivating tasks, promotion-oriented job crafters experience their work activities in a personally meaningful way (Tims and Bakker, 2010; Wrzesniewski et al., 2013) and report higher levels of perceived meaningfulness at work (Tims et al., 2016). In addition, by seeking job resources, employees can expand their resource pool, which boosts their work engagement and protects them from exhaustion (Bakker et al., 2005). This is because job resources enhance intrinsic motivation and provide tools to reduce the costs of dealing with job demands (Demerouti et al., 2001). In fact, Tims et al. (2013) found that seeking resources leads to an actual increase in perceived job resources, which leads to heightened work engagement and reduced burnout, of which exhaustion is one component.

Contrary to promotion-oriented job crafting, prevention-oriented job crafting often fails to produce favorable changes in tangible work role boundaries (e.g., no decreases in hindering job demands). Prevention-oriented job crafting may protect employees well-being, however, employees also reduce the triggers or necessity for action (Petrou et al., 2012), in other words, the optimal level of job challenge (Csikszentmihalyi, 1990) in their daily activities. Employees’ time and effort investment in prevention-oriented job crafting may not be fruitful over time, leading to accumulating workloads and subsequent backlash, which makes the work more exhausting (Tims et al., 2015). According to COR, the negative emotional experience is regarded as a loss of resources. At this time, employees will start to protect their existing resources and reduce engagement, then they may fall into a spiral of resource depletion (Lazarus, 1991). For example, prevention-oriented job crafters may perceive their job as less meaningful, experiencing less coherence at work (Lichtenthaler and Fischbach, 2019). Thus, we propose the following hypotheses:

*Hypothesis 2a:* Promotion-oriented job crafting is positively related to work meaning.

*Hypothesis 2b:* Promotion-oriented job crafting is negatively related to emotional exhaustion.

*Hypothesis 2c:* Prevention-oriented job crafting is negatively related to work meaning.

*Hypothesis 2d:* Prevention-oriented job crafting is positively related to emotional exhaustion.

Turnover intention reflects an individual’s likelihood of quitting their job (Konovsky and Cropanzano, 1991). While turnover intention is usually considered the ultimate outcome, emotional exhaustion and work meaning have been reported to mediate the effect of job crafting on employees’ intention to leave (Shin et al., 2020). Employees who find their work meaningful are inclined to stay longer in their current position, perceiving their jobs as more meaningful (Oprea et al., 2022). When threatened with resource loss, exhausted individuals often use withdrawal as a coping mechanism to prevent further loss of resources and psychological costs of exhaustion (Hobfoll, 1989). As emotionally exhausted employees feel reluctant to spend additional resources on their job, they choose to reduce their commitment to the organization and start to look for new jobs (Thanacoody et al., 2014).

In sum, employees engage in promotion-oriented job crafting to craft motivating job characteristics (such as job resources and challenging job demands) to their needs and abilities. Through such crafting, they often perceive their work as more meaningful and experience positive emotions, leading to a reduced likelihood of turnover intention. Conversely, employees adopting prevention-oriented job crafting may not always successfully eliminate challenging job characteristics. Consequently, they might find their work less meaningful, experience negative emotions more frequently, and face an increased risk of turnover intention. Based on findings from previous studies, we propose the following hypotheses:

*Hypothesis 3a:* Work meaning and emotional exhaustion mediate the negative relationship between promotion-oriented job crafting and turnover intention.

*Hypothesis 3b:* Work meaning and emotional exhaustion mediate the positive relationship between prevention-oriented job crafting and turnover intention.

#### The interaction between promotion- and prevention-oriented job crafting and their effects

As discussed before, an ambidexterity framework suggests a cyclic and interactive relationship between promotion and prevention focuses. Individuals who simultaneously pursue promotion- and prevention-oriented job crafting are more effective than those who only engage in promotion-oriented or prevention-oriented job crafting(Cegarra-Navarro et al., 2011), because individuals who aim to both exploit and explore their job need to develop cognitive schemas that accommodate both efficiency-oriented and variability-increasing goals (Bonesso et al., 2014). Individuals can recognize the contradictions inherent to different tasks while understanding the relationship among these tasks as complementary or reinforcing, thus achieving their desired career goals. They view prevention-oriented job crafting as a health factor, which protects personal resources from being lost. At the same time, they adopt promotion-oriented job crafting to acquire more resources. Through the spiral increase of resources, it has a positive impact on individuals.

Recent research has examined how approach and avoidance forms of job crafting interact with each other (Petrou and Xanthopoulou, 2020). Building upon this, it is reasonable for us to argue that just like the independent job crafting forms, the interaction between prevention- and promotion-oriented job crafting could significantly influence employees’ turnover intention through other factors. Based on existing literature, we hypothesize that:

*Hypothesis 4a*: Work meaning and emotional exhaustion mediate the negative relationship between the interaction between promotion- and prevention-oriented job crafting (ProJC*PreJC) and turnover intention.

*Hypothesis 4b*: Work meaning and emotional exhaustion mediate the negative relationship between the difference between promotion- and prevention-oriented job crafting (ProJC-PreJC) and turnover intention.

## Study 1: scale development

Based on the theoretical model proposed in the previous section, the goal of the first study is to develop and validate a scale for measuring employees’ ambidextrous job crafting. We first describe the process of item generation and selection and then present the exploratory and confirmatory factor structures of the ambidextrous job crafting scale as well as its reliability test result.

### Method

#### Procedure and participants

A total of 1,424 employees participated in Study 1. First, 124 MBA/MPA students (Sample 1.1) completed an open-ended questionnaire about “How to make yourself feel better by actively changing the work?” under different circumstances. Their responses were later used to construct the new ambidextrous job crafting scale. Of the 124 respondents, we received 118 valid responses, representing a response rate of 95.16%. Specifically, our participants were mostly male (57.3%) and worked in a nonmanagerial position (63.4%). All of them were full-time employees from various companies and government sectors who attended weekend classes for 3 years to earn their master’s degrees.

A second sample (Sample 1.2) is used to validate the scale. To maximize the sampling efficiency and the sample coverage, we recruited respondents through the researcher’s social network using a combination of convenient and snowball sampling methods. The final pool included 600 respondents from 11 organizations located in two provinces in China, namely Beijing and Henan. In total, we received 344 valid responses, representing a response rate of 57.33%. Participants were predominantly males (60.5%), relatively young (mean age = 35.24, *SD* = 8.59), and well-educated (70% had a college or higher degree). On average, our participants have been working for their current employers for 8.78 (*SD* = 8.55) years; the majority (67.2%) of them worked in a non-managerial position, 58.7% worked in state-owned enterprises (SOEs), 29.3% worked in non-SOEs (private firms and joint ventures), and 11.9% worked in government sectors or public institutions. In addition, nearly half (49.6%) of the employees worked for large firms with more than 500 employees, while about half (50.9%) of the sample worked in firms at the mature and revitalization stage.

Sample 1.3 is used to examine whether the proposed factor structure can be reliably replicated in a different sample using confirmatory factor analysis. It is also a convenience sample recruited through the researcher’s social network. Participants were employees from 13 enterprises located in five provinces in China, including Beijing, Hebei, Henan, Tianjin, and Shandong. A total of 461 copies of valid responses were received. Specifically, our participants were primarily female (62.5%), relatively young (mean age = 34.65, *SD* = 8.44), and well-educated (73% had a college or higher degree). On average, participants have been working 8.55 (*SD* = 8.64) years for their current employers; the majority (68.1%) of the sample worked in a non-managerial position. 63.8% in SOEs, 24.1% in non-SOEs, and 12.4% in government sectors or public institutions. In addition, nearly half (48.1%) of the employees worked for large firms with more than 500 employees. 55.1% of these employees worked in firms at the mature and revitalization stage.

#### Item generation

Based on previous job crafting measurements, the results of our open-ended survey, and expert evaluation, a total of 92 items were generated to measure ambidextrous job crafting comprehensively. First, we analyzed responses to the open-ended survey (Sample 1.1) and created 74 representative ambidextrous job crafting measurements. Sample responses include: “think about work from the perspective of organizational development” “share resources with colleagues proactively” (promotion-focus); and “reduce the amount of work that I am not interested in” “avoid making decisions when I am emotionally agitated” (prevention focuses).

Then, an extensive review of the literature concerning the measurement of the “ambidextrous” nature of job crafting was conducted, and 122 relevant items were selected from well-established scales such as the job crafting scale (Tims et al., 2012), the Job Crafting Questionnaire (Bindl et al., 2019), and the Job Crafting Scale (Weseler and Niessen, 2016). We selected these items based on the features proposed by March (1991) to describe the constructs of exploration and exploitation. We also referred to previous studies illustrating individuals’ ambidextrous behaviors regarding exploration- and exploitation-related activities during the item selection process (e.g., Mom et al., 2009; Kauppila and Tempelaar, 2016). The selected items can thus be considered as a group of valid measurements representative of the preestablished measurements of ambidexterity in job crafting.

Lastly, a team of organizational behavior experts was invited to examine the similarities and differences among items. After several rounds of item reduction, 92 items- 73 available items and 19 new items–with satisfying levels of parsimony, theoretical consistency, and functionality were kept for a new ambidextrous job crafting scale with a 10-factor structure. Among the 92 items, there are 9 items on promotion-oriented cognitive crafting (Pro-CC); 10 items on prevention-oriented cognitive crafting (Pre-CC); 9 items on promotion-oriented emotional crafting (Pro-EC); 8 items on prevention-oriented emotional crafting (Pre-EC); 9 items on promotion-oriented skill crafting (Pro-SC); 8 items on prevention-oriented skill crafting (Pre-SC); 9 items on promotion-oriented task crafting (Pro-TC); 9 items on prevention-oriented task crafting (Pre-TC); 13 items on promotion-oriented relationship crafting (Pro-RC); and 8 items on prevention-oriented relationship crafting (Pre-RC).

#### Analysis techniques

In this study, reliability analysis, skewness and kurtosis analysis, internal consistency criterion analysis, total item correlation, and exploratory factor analysis (EFA) were calculated using SPSS 18.0. Confirmatory factor analyses (CFAs) were conducted using AMOS Version 23.0. We evaluated the model fit using five indices: *χ^2^* per degree of freedom (*χ^2^/df*), the normed fit index (NFI), the comparative fit index (CFI), the Tucker-Lewis index (TLI), and the root mean square error of approximation (RMSEA). Generally, a model is considered to have an acceptable fit if *χ^2^/df* is equal to or lower than 5 (Bollen, 1989), NFI, CFI, and TLI values are above 0.90, and the RMSEA is between 0.05 and 0.08 (Browne and Cudeck, 1992; Hu and Bentler, 1999).

### Results and discussion

#### Item analysis

We administered the 92 ambidextrous job crafting items to respondents (Sample 1.2) to explore the factor structure of the ambidextrous job crafting scale. We asked respondents to evaluate if the statements fit their current or past work behaviors, using a 5-point Likert-type scale (1 = *strongly disagree*, 5 = *strongly agree*).

We first analyzed the internal consistency, and 9 items were excluded due to their negative influence on Cronbach’s Alpha value. Next, we conducted an item discrimination analysis. We calculated each participant’s total score for the remaining 83 items and extracted two groups from our sample according to their scores. Those who ranked in the top 27% were categorized into the high-score group, while those who ranked in the bottom 27% were categorized into the low-score group. For each item, we calculated its mean for the high-score and low-score groups, respectively, and conducted a t-test to see whether there was a significant (*p* < 0.05) difference between the high versus low mean item scores. As a result, 83 items with significant critical ratio values - meaning that the item could differentiate the reactions between the high-score and low-score groups - were retained. In addition, we conducted a normality test by observing the skewness and kurtosis values, then examined each item’s correlation with its dimension mean. To ensure the content validity of our measurement items, we did not delete any item until after repeated tests and discussions regarding its applicability. At last, 83 items of the original 92 items were retained.

#### Exploratory factor analysis

To examine whether the newly constructed scale followed the 10 theoretical dimensions of ambidextrous job crafting, we examined the factor structure and reliability of the scale with exploratory principal component analyses (PCAs) using the remaining 83 items (Sample 1.2). Items were dropped if they had loadings less than 0.40 or cross-loaded on multiple factors with loadings greater than 0.40. This procedure was repeated until the factor solution satisfied all criteria specified above. Based on the items analysis, exploratory factor analysis, and expert discussion results, 42 potentially confusing or redundantly worded items were excluded. This culling process resulted in 41 items to measure ambidextrous job crafting in the organization context (see Appendix A). As expected, a 10-factor structure was identified by the EFA model. Each factor consisted of three to five items, with loading values ranging from 0.54 to 0.86.

Specifically, the 10 factors explained 62.44% of the variance. The first three factors–Pro-SC, Pre-EC, and Pre-SC–included five items each; the following five factors–Pre-TC, Pre-CC, Pre-RC, Pro-CC, and Pro-TC - included four items each; the last two factors - Pro-EC and Pro-RC - included three items each. All 10 factors demonstrated good reliability, with Cronbach’s alphas ranging from 0.68 to 0.87.

#### Confirmatory factor analysis

The 41 items selected from the previous procedures were used for the measurement of ambidextrous job crafting (Sample 1.3). Cronbach’s alphas were above the 0.70 threshold recommended by Nunnally and Bernstein (1994) for all 10 factors, ranging from 0.720 for Pre-RC to 0.869 for Pro-SC.

To test Hypothesis 1, whether the 10-factor solution also fits best in the new sample, we fitted a series of CFA models using sample 1.3. We also compared the proposed model (Model 7 [M7]) with six possible alternative models (Models 1–6). The results showed that M7 showed an acceptable model fit (*χ^2^/df* = 2.354; NFI = 0.769, IFI = 0.853, TLI = 0.842, CFI = 0.852, RMSEA = 0.054), and was significantly better than other alternative models (Table 1). According to Figure 1, promotion- and prevention-oriented job crafting factors were moderately correlated (mean corrected correlation = 0.43); all items also demonstrated sufficient loadings on their associated factors, ranging from 0.40 to 0.85.

**Table 1:** Fit indices of the models (*N* = 461).

Model	χ^2^	df	χ^2^/df	Δχ^2^	RMSEA	NFI	IFI	TLI	CFI
Model 1	4712.259	779	6.049	264.007**	0.105	0.399	0.443	0.410	0.439
Model 2	4535.630	778	5.830	272.745**	0.102	0.421	0.467	0.435	0.464
Model 3	4505.368	778	5.791	269.719**	0.102	0.425	0.472	0.440	0.469
Model 4	3964.658	776	5.109	269.560**	0.095	0.494	0.548	0.520	0.545
Model 5	3762.131	776	4.848	244.244**	0.091	0.520	0.577	0.550	0.574
Model 6	3059.403	769	3.978	1251.221**	0.080	0.609	0.676	0.652	0.673
Model 7	1808.182	768	2.354		0.054	0.769	0.853	0.842	0.852

Model 1 denoted that a single first-order factor, Ambidextrous Job Crafting, accounts for the 41 items; Model 2 merged cognitive, skill, task, and relationship crafting in M3 as one factor; Model 3 suggested that the 19 observable promotion-oriented items and the 22 observable prevention-oriented items loaded on their respective factors; Model 4 merged cognitive and emotional crafting in M3 as one factor; Model 5 merged cognitive, task, and relationship crafting in M3 as one factor; Model 6 showed that the 41 observable items were accounted for by five first-order factors (cognitive, emotional, skill, task, and relationship crafting); Model 7denoted that the 41 observable items loaded on the ten first-order factors (promotion- and prevention-oriented cognitive, emotional, skill, task, and relationship crafting), which then loaded on the second-order factors (promotion- and prevention-oriented job crafting). **p < 0.01; NFI = normed fit index; TLI = Tucker–Lewis Index; CFI = comparative fit index; RMSEA = root mean square error of approximation.

In study 1, we developed and confirmed the ambidextrous job crafting scale using both quantitative and qualitative methods. As we hypothesized, the 41 theoretically and empirically generated items loaded satisfyingly on 10 factors belonging to five dimensions of two intercorrelated job crafting orientations and eventually loaded on a single overarching factor: ambidextrous job crafting. Considering that the items were selected based on our theoretical framework of ambidexterity, it is reasonable for us to argue that the results provide preliminary evidence for the structural validity of our ambidextrous job crafting scale.

## Study 2: the effects of ambidextrous job crafting

In Study 2, we further examined the ambidextrous nature of job crafting by testing the connection between the results of our ambidextrous job crafting scale and various work outcomes and other scales; we also examined the scale’s test–retest reliability.

### Method

#### Procedure and participants

Sample 2 is a convenience sample collected from a state-owned enterprise in Henan province, China. In total, 659 employees from three departments filled out this questionnaire. To avoid the common method variance, a time-lagged method was employed to test the effects of ambidextrous job crafting. At Time 1, participants reported their levels of ambidextrous job crafting as well as demographic information. At Time 2 (12 days after Time 1), participants were asked to report their perceptions of work meaning and emotional exhaustion. At Time 3 (12 days after Time 2), participants were asked to report their ambidextrous job crafting again together with their turnover intention. After matching the cases from Time 1, Time 2, and Time 3, we obtained the final sample of 387 valid cases, with a response rate of 58.7%. Overall, the majority of participants from Sample 2 were males (88.1%), relatively young (mean age = 36.03, *SD* = 10.14), and less educated than the previous samples (39.3% had a college or higher degree). On average, participants of this sample had been working for 13.39 years (*SD* = 11.47) for their current employer.

#### Measures

##### Ambidextrous job crafting

Ambidextrous job crafting was measured by the 41-item scale developed in our previous study. As suggested by the CFA results, the second-order structure demonstrated an acceptable fit in the current analysis (*χ^2^* = 2133.010, *df* = 768, *χ^2^/df* = 2.777, CFI = 0.793, TLI = 0.779, NFI = 0.712, IFI = 0.794, RMSEA = 0.068). The Cronbach’s alpha values ranged from 0.628 for Pre-EC to 0.803 for Pro-SC. The overall Cronbach’s *α* was 0.831. For each sub-scale of the ambidextrous job crafting scale - i.e., the promotion- and prevention-scale - we calculated its mean as the scale score. Following previous studies (e.g., He and Wong, 2004; Cao et al., 2009), we calculated the relative difference between the promotion- and prevention-oriented job crafting strategy scores (ProJC-PreJC) to represent the ambidexterity between the two. Since prevention-oriented job crafting functions as a protective factor, a larger difference indicates that while holding the prevention score constant, employees who can better switch between the two types of job crafting score higher on promotion dimension, leading to a higher level of ambidexterity. Similarly, we calculated the product of the two scores (ProJC*PreJC) to represent the combined importance of the two, with a higher product score indicating employee’s stronger emphasis on both dimensions. Taken together, the product score (ProJC*PreJC) and the relative difference score (ProJC-PreJC) represent employees’ level of ambidextrous job crafting.

##### Job crafting

We also examined the incremental validity of ambidextrous job crafting, i.e., its superiority compared to alternative forms of job crafting. Job crafting was assessed with the job crafting scale (JCA) developed by Tims et al. (2012). The scale consists of 21 items that cover four dimensions. To reduce the survey time and increase the effective response rate (Rolstad et al., 2011), we created an abbreviated JCA by including only the two items with the highest loadings on each JCA dimension. Items selected from the four dimensions include: “I try to develop my capabilities” and “I try to develop myself professionally” (increasing structural job resources); “I ask my supervisor to coach me” and “I ask whether my supervisor is satisfied with my work” (increasing social job resources); “When an interesting project comes along, I offer myself proactively as project co-worker” and “If there are new developments, I am one of the first to learn about them and try them out” (increasing challenging job demands); “I make sure that my work is mentally less intense” and “I try to ensure that my work is emotionally less intense” (decreasing hindering job demands). In this study, all items are scored on a 5-point Likert-type scale (1 = *absolutely wrong*, 5 = *absolutely true*). The Cronbach’s alpha was 0.833 for the full scale.

##### Work meaning

Work meaning was measured using the 10-item scale developed by Steger et al. (2012). The scale contains three subscales - Positive Meaning (PM), Meaning Making through Work (MM), and Greater Good Motivations (GGM) - with three to four items for each subscale; a higher score indicates a stronger sense of work meaning. In the current study, employees were asked to consider a subjectively meaningful experience consisting of experiencing positive meaning in work, sensing that work is a key avenue for making meaning, and perceiving one’s work to benefit some greater good. One of the ten original items (“My work really makes no difference to the world (R).”) demonstrated low factor loading and substantially decreased the psychometric qualities of the measure, thus was deleted from the GGM. Example items for the three dimensions are as follows: “I have found a meaningful career” (PM, *α* = 0.818); “My work helps me better understand myself” (MM, α = 0.843); and “The work I do serves a greater purpose” (GGM, α = 0.752). The overall Cronbach’s α for the full scale was 0.898. All the items were rated on a 5-point Likert scale (1 = *absolutely wrong*, 5 = *absolutely true*).

##### Emotional exhaustion

Emotional Exhaustion was measured using the 9-item scale developed by Maslach and Jackson (1981). The nine items in the Emotional Exhaustion subscale describe feelings of being emotionally overextended and exhausted by one’s work. A sample item is “I feel I’m working too hard on my job.” The Cronbach’s alpha was 0.946 for the full scale. All items were rated on a 7-point Likert scale (1 = *very mild, barely noticeable*, 7 = *major, very strong*).

##### Turnover intention

Turnover intention was measured using the 3-item scale developed by Konovsky and Cropanzano (1991). An example item is “I will look for a job outside this organization during the next year.” The Cronbach’s alpha was 0.885 for the full scale. A five-point Likert scale was used (1 = *strongly disagree*, 5 = *strongly agree*).

#### Analysis techniques

In this study, descriptive statistics, test–retest reliability, and discriminant and predictive validity were calculated using SPSS 18.0. Convergent validity and path analysis results were calculated using AMOS 23.0.

### Results and discussion

#### Descriptive statistics, and discriminant and convergent validation

Descriptive statistics and correlations of the main variables in Study 2 are displayed in Table 2. The correlation analysis showed that promotion-oriented job crafting had a significant positive correlation with work meaning (*r* = 0.504, *p* < 0.01) and negative correlations with emotional exhaustion (*r* = −0.203, *p* < 0.01) and turnover intention (*r* = −0.166, *p* < 0.01). Prevention-oriented job crafting showed significant positive correlations with emotional exhaustion (*r* = 0.338, *p* < 0.01) and turnover intention (*r* = 0.316, *p* < 0.01). Although insignificant, the correlation between prevention-oriented job crafting and work meaning was negative (*r* = −0.077, *p >* 0.05). These results confirmed the convergent validity of the five variables.

**Table 2:** Descriptive statistics and correlations among variables in study 2 (*N* = 387).

Variable	M	SD	1	2	3	4	7	8	9
Promotion-oriented job crafting	4.372	0.475	–						
Prevention-oriented job crafting	3.356	0.462	0.040	–					
ProJC-PreJC	1.017	0.649	0.703^**^	−0.683^**^	–				
ProJC*PreJC	14.679	2.691	0.617^**^	0.808^**^	−0.124^*^	–			
Job crafting	4.408	0.502	0.544^**^	−0.019	0.411^**^	0.303^**^	–		
Work meaning	4.405	0.560	0.504^**^	−0.077	0.423^**^	0.230^**^	0.656^**^	–	
Emotional exhaustion	2.049	0.959	−0.203^**^	0.338^**^	−0.390^**^	0.148^**^	−0.345^**^	−0.516^**^	-
Turnover intention	1.978	1.048	−0.166^**^	0.316^**^	−0.346^**^	0.153^**^	−0.236^**^	−0.430^**^	0.597^**^

ProJC: Promotion-oriented Job Crafting; PreJC: Prevention-oriented Job Crafting; ^**^p < 0.01, ^*^p < 0.05.

We then conducted a CFA to compare the measurement models and test the discriminant validity of the five variables. In addition to the benchmark model (Model 1 [M1]: five-factor model), we provided four alternative models (Models 2–5). The results showed that the other alternative models were significantly worse than the benchmark model (M1: *χ^2^/df =* 2.290, CFI = 0.826, TLI = 0.818, RMSEA = 0.065; Table 3). Taken together, ambidextrous job crafting scale has good convergent and discriminant validity.

**Table 3:** Comparisons of measurement models (*N* = 387).

Model	χ^2^	df	χ^2^/df	△χ^2^	NFI	IFI	TLI	CFI	RMSEA
Model 1	4142.783	1809	2.290		0.729	0.827	0.818	0.826	0.065
Model 2	5251.106	1823	2.880	79.166**	0.656	0.745	0.734	0.744	0.070
Model 3	6340.150	1826	3.472	129.257**	0.585	0.664	0.651	0.663	0.080
Model 4	6790.627	1828	3.715	139.360**	0.556	0.631	0.617	0.629	0.084
Model 5	9568.841	1829	5.232	271.303**	0.374	0.424	0.402	0.422	0.105

M1 included promotion-oriented job crafting, prevention-oriented job crafting, work meaning, emotional exhaustion, and turnover intention. Model 2 (M2) merged emotional exhaustion and turnover intention in M1 as one factor, Model 3 (M3) merged promotion- and prevention-oriented job crafting and work meaning in M1 as one factor, Model 4 (M4) merged emotional exhaustion and turnover intention in M3 as one factor, Model 5 (M5) merged the five variables into one factor. **p < 0.01; NFI = normed fit index; TLI = Tucker–Lewis Index; CFI = comparative fit index; RMSEA = root mean square error of approximation.

#### Predictive validity

The correlation analysis showed that the difference variable “ProJC-PreJC” was significantly and positively correlated with work meaning (*r* = 0.423, *p* < 0.01) while significantly and negatively correlated with emotional exhaustion (*r* = −0.390, *p* < 0.01) and turnover intention (*r* = −0.346, *p* < 0.01). The product variable “ProJC*PreJC” had significant positive correlations with work meaning (*r* = 0.230, *p* < 0.01), emotional exhaustion (*r* = 0.148, *p* < 0.01), and turnover intention (*r* = 0.153, *p* < 0.01). Table 2 summarizes these results.

We also examined whether ambidextrous job crafting had incremental predictive validity relative to job crafting. As shown in Table 4, after all the control variables and job crafting were added to the equation, “ProJC-PreJC” was still significantly correlated with work meaning (*β* = 0.185, *p* < 0.01), emotional exhaustion (*β* = −0.332, *p* < 0.01), and turnover intention (*β* = −0.307, *p* < 0.01). “ProJC*PreJC” was still significantly correlated with emotional exhaustion (*β* = 0.288, *p* < 0.01) and turnover intention (*β* = 0.253, *p* < 0.01). Although not significant, the effect of “ProJC*PreJC” on work meaning was positive (*β* = 0.041, *p* > 0.05). Overall, the findings showed that the ambidextrous job crafting scale has incremental predictive validity.

**Table 4:** Incremental regression analysis of AJC on work meaning, emotional exhaustion, and turnover intention (*N* = 387).

Variable	Work meaning			Emotional exhaustion			Turnover intention		
	Step 1	Step 2	Step 3	Step 1	Step 2	Step 3	Step 1	Step 2	Step 3
Tenure	0.028	0.040	0.027	0.103	0.081	0.095	−0.040	−0.060	−0.047
Position	0.032	0.000	0.034	0.008	0.066	0.021	−0.065	−0.011	−0.053
Age	0.041	0.040	0.039	−0.124	−0.123	−0.136	−0.043	−0.042	−0.053
Gender	−0.007	0.000	−0.010	0.004	−0.010	−0.018	0.083	0.071	0.064
Job crafting	0.657^**^	0.581^**^	0.645^**^	−0.348^**^	−0.212^**^	−0.435^**^	−0.244^**^	−0.118^*^	−0.321^**^
ProJC-PreJC		0.185^**^			−0.332^**^			−0.307^**^	
ProJC*PreJC			0.041			0.288^**^			0.253^**^
R^2^	0.436	0.463	0.437	0.129	0.217	0.203	0.076	0.151	0.134
△R^2^	0.436^**^	0.027^**^	0.002	0.129^**^	0.088^**^	0.074^**^	0.076^**^	0.075^**^	0.057^**^

**P**roJC: Promotion-oriented Job Crafting; PreJC: Prevention-oriented Job Crafting; Tenure:1 ≤ 10 years, 2 > 10 years; position:1 = employee, 2 = manager; age: 1 ≤ 30 years, 2: 31 < age≤40 years, 3 > 10 years; gender:1 = female, 2 = male. ^*^p < 0.05. ^**^p < 0.01.

#### Test–retest reliability

We examined the ambidextrous job crafting scale’s test–retest reliability between Time 1 and Time 3, which were 24 days apart. Both job crafting scores at Time 1 were significantly correlated with their corresponding scores at Time 3 (*r*-promotion = 0.543, *r*-prevention = 0.502, *p* < 0.01), indicating that the scale of ambidextrous job crafting has sufficient test–retest reliability.

#### Path analysis results

Lastly, we conducted path analyses with robust maximum likelihood estimation to test Hypotheses 2 through 4.

##### Results of promotion- and prevention-oriented job crafting

**Figure 3:**
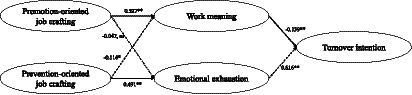
Promotion- and prevention-oriented job crafting on turnover intention: the dual-path effects.

Results of structural equation models are shown in Figure 3. We compared two models: Model 1 (M1, the hypothesized model) was the fully mediated model with work meaning and emotional exhaustion being the mediators, while Model 2 (M2, the alternative nested model) added two direct paths from promotion- and prevention-oriented job crafting to turnover intention through work meaning and emotional exhaustion, respectively. The results showed that M1 (*χ^2^/df* = 2.345, NFI = 0.722, IFI = 0.819, TLI = 0.810, CFI = 0.818, RMSEA = 0.059) and M2 (*χ^2^/df* = 2.341, NFI = 0.723, IFI = 0.820, TLI = 0.811, CFI = 0.819, RMSEA = 0.059) both showed acceptable model fits, while M2 was not superior to M1 [△*χ^2^*(2)/*df* = 5.273, *p* > 0.05]. Therefore, adding direct paths from promotion- and prevention-oriented job crafting to turnover intention could not significantly improve the fit indices of the model.

As shown by the structural equation model (Figure 3), promotion-oriented job crafting significantly and positively predicted work meaning (*β* = 0.527, *p* < 0.01). Prevention-oriented job crafting significantly and positively predicted emotional exhaustion (*β* = 0.491, *p* < 0.01), but it significantly and negatively predicted work meaning (*β* = −0.114, *p* < 0.05). Work meaning significantly and negatively predicted turnover intention (*β* = −0.139, *p* < 0.01), and emotional exhaustion significantly and positively predicted turnover intention (*β* = 0.616, *p* < 0.01).

The bias-corrected bootstrap test was used to further examine the size and confidence intervals of work meaning and emotional exhaustion’s mediating effect with a sampling size of 5,000 and a confidence interval of 95%. The results indicated that the indirect effect of promotion-oriented job crafting through work meaning was significant because the 95% CI ([−0.745, −0.393]) did not contain zero; in contrast, the indirect effect for emotional exhaustion was not significant. The indirect effect of prevention-oriented job crafting through emotional exhaustion (95% CI [0.233, 0.446]) and work meaning (95% CI [0.096, 0.248]) was significant. Thus, Hypothesis 2a, 3a, 3b are supported, and Hypothesis 2b is partially supported.

##### Results of “ProJC-PreJC”

**Figure 4:**
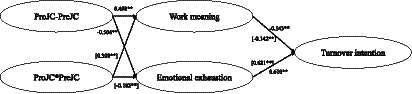
The balance and interaction between promotion- and prevention-oriented job crafting on turnover intention: the dual-path effects.

The structural equation model is shown in Figure 4. We compared two models: Model 3 (M3, the hypothesized model) was a fully mediated model, while Model 4 (M4, the alternative nested model) added two direct ways from “ProJC -PreJC” to turnover intention through work meaning and emotional exhaustion, respectively. The results showed that M3 (*χ^2^/df* = 3.623, NFI = 0.854, IFI = 0.890, TLI = 0.878, CFI = 0.889, RMSEA = 0.082) and M4 (*χ^2^/df* = 3.621, NFI = 0.854, IFI = 0.890, TLI = 0.878, CFI = 0.890, RMSEA = 0.082) both demonstrated an acceptable model fit. M4 was not superior to M3 judging by the fit indices (△*χ^2^*(1)/*df* = 4.042, *p* < 0.05). The results suggested that adding direct paths from “ProJC -PreJC” to turnover intention could significantly improve the fit indices of the model, but the effect of “ProJC -PreJC” job crafting on turnover intention was not significant (*β* = −0.112, *p* > 0.05). Therefore, M3 is superior to M4.

According to the structural equation model (Figure 4), it is clear that “ProJC- PreJC” significantly and positively predicted work meaning (*β* = 0.498, *p* < 0.01) and negatively predicted emotional exhaustion (*β* = −0.504, *p* < 0.01). Work meaning significantly and negatively predicted turnover intention (*β* = −0.143, *p* < 0.01), and emotional exhaustion significantly and positively predicted turnover intention (*β* = 0.610, *p* < 0.01). The results indicated that the indirect effect of “ProJC-PreJC” through work meaning (95% CI [−0.279, −0.139]) and emotional exhaustion (95% CI [−0.387, −0.204]) was significant. Thus, Hypothesis 4a is supported.

##### Results of “PreJC*ProJC”

The structural equation model is shown in Figure 4. We compared two models: Model 5 (M5, the hypothesized model) was a fully mediated model, while Model 6 (M6, the alternative nested model) added two direct ways from “PreJC*ProJC” to turnover intention through work meaning and emotional exhaustion, respectively. The results showed that M5 (*χ^2^/df* = 4.033, NFI = 0.830, IFI = 0.867, TLI = 0.852, CFI = 0.866, RMSEA = 0.089) and M6 (*χ^2^/df* = 4.043, NFI = 0.830, IFI = 0.867, TLI = 0.852, CFI = 0.866, RMSEA = 0.089) both demonstrated an acceptable model fit. M6 was not superior to the M5 (△*χ^2^*(1)/*df* = 1.104, *p >* 0.05). The results showed that adding direct paths from “PreJC*ProJC” to turnover intention could not significantly improve the fit indices of the model.

According to Figure 4, it is clear that “PreJC*ProJC” could significantly and positively predict work meaning (*β* = 0.308, *p* < 0.01) and could negatively predict emotional exhaustion (*β* = −0.182, *p* < 0.01). Work meaning significantly and negatively predicted turnover intention (*β* = −0.142, *p* < 0.01), and emotional exhaustion significantly and positively predicted turnover intention (*β* = 0.621, *p* < 0.01). The results indicated that the indirect effect of “PreJC*ProJC” through work meaning (95% CI [0.001, 0.039]) and emotional exhaustion (95% CI [0.052, 0.112]) was significant. Thus, Hypothesis 4b is supported.

Using a lagged study design, we confirmed the test–retest reliability of the ambidextrous job crafting scale in a separate sample. We also found that ambidextrous job crafting, operationalized as the interaction and the relative difference between prevention and promotion crafting strategies, has sufficient convergent and predictive validity. According to the path analysis results, ambidextrous job crafting was negatively related to turnover intention through work meaning and emotional exhaustion. This result is consistent with previous studies (Caniëls and Veld, 2019).

However, we found that the effect of promotion-oriented job crafting on emotional exhaustion was insignificant. Using self-regulation and role theories, we argue that promotion-oriented job crafting requires additional effort, which may come at the cost of energy depletion (Bakker and Oerlemans, 2019). Promotion focused coping entails cognitive, emotional, and behavioral efforts that maximize the chances for a match between one’s current situation and one’s hopes and aspirations. These job crafting strategies require substantial energy, which may deplete employees’ energy reservoirs and make feel used up and worn out.

## Discussion

Previous job crafting literature has typically viewed prevention- and promotion-oriented job crafting as distinct strategies associated with different work outcomes and activities. Our findings, however, suggest that these two orientations do not lie at opposite ends of a spectrum. Instead, both are linked to exploitative and explorative activities and are often adopted simultaneously by employees. Therefore, we introduced the concept of ambidexterity into regulatory focus theory and Wrzesniewski and Dutton’s (2001) theoretical model, arguing that ambidextrous job crafting should be incorporated to better capture the complexity of employees’ job crafting behaviors. Informed by this perspective, we addressed the issue of the negative effects that may arise from the prevention-oriented job crafting (Lichtenthaler and Fischbach, 2019). Furthermore, we developed a novel ambidextrous job crafting scale using both quantitative and qualitative methods. Data collected from major Chinese cities suggested that the 41-item scale has good validity, test–retest reliability, incremental validity and predictive validity for measuring employees’ ambidextrous job crafting.

### Theoretical implications

First, this study contributes to the job crafting literature by introducing the concept of ambidextrous job crafting and developing a validated measurement scale. Future research is needed to further elucidate how these dimensions interrelate. By integrating the regulatory focus perspective, we reveal the inherent ambidexterity in job crafting and emphasize the dynamic, interdependent relationship between promotion and prevention focuses. Unlike most prior studies that treated these focuses as independent strategies (Bindl et al., 2019; Lichtenthaler and Fischbach, 2019), our approach acknowledges that ambidextrous job crafting involves employees belding promotion-related and prevention-related activities, thus leveraging their synergistic potential. Through the integration and the flexible switch between promotion- and prevention-oriented job crafting, the two opposites coexist harmoniously and interdependently, forming a continually evolving and transforming whole. This novel interpretation significantly enriches the general understanding of the ambidextrous nature of job crafting.

Second, this study expands understanding of job crafting’s effects by uncovering dual-path mechanisms through which promotion- and prevention-oriented job crafting differentially influence turnover intention. While the theory posits a positive correlation among its four dimensions - increasing structural job resources, increasing challenging job demands, increasing social job resources, and decreasing hindering job demands - due to their shared focus on proactive work environment modification, the dimension of decreasing hindering job demands has showed varied relationships (Tims et al., 2012). Our study explores the complex mediating mechanism by integrating the COR theory, examining how resources are expended or conserved during employees’ engagement in ambidextrous job crafting. Additionally, our findings confirm the positive effects of ambidextrous job crafting on career and wellbeing outcomes, such as work meaning, emotional exhaustion, and turnover intention. This extends the analytic framework of ambidextrous job crafting to encompass crucial work-related outcomes.

Third, this study advances the ambidexterity literature by operationalizing the interaction between promotion and prevention foci. We introduce a novel operationalization of ambidextrous job crafting in the job crafting literature, focusing on the interaction between promotion and prevention processes, as indicated by their relative differences and combined effect. This approach offers new insights into the ambidexterity construct, dissecting individual processes to understand how various job crafting strategy combinations affect individual turnover intention. Our findings support our hypotheses by demonstrating distinct relationships between job crafting strategies, work meaning, and emotional exhaustion. Both the difference score and the product score between promotion- and prevention-oriented job crafting were found to positively correlate with work meaning and negatively with emotional exhaustion. Notably, the correlation between the difference score and the outcomes was stronger than that of the product score. These results suggest that a balanced “ProJC-PreJC” strategy could be more effective for individuals, not only in resolving resource conflicts and contradictory demands but also in enabling prevention-oriented job crafting to serve as a protective health factor (Tims et al., 2012).

### Practical implications

We would also like to note the methodological and practical implications of the current study. First, by employing both quantitative and qualitative methods to explore the construct of ambidextrous job crafting, this study responds to calls for deeper investigation into emotional crafting (Tian, 2022). Although emotions have been implicitly acknowledged in previous job crafting research (Tims et al., 2012; Lazazzara et al., 2020), most studies have either primarily focused on the rational aspects of crafting behaviors (Bruning and Campion, 2018), treating emotions as secondary to rational processes; or the dimension of emotional reconfiguration has been theoretically proposed, corresponding measurement tolls remain lacking (Eguchi et al., 2016). By integrating role (Wrzesniewski and Dutton, 2001; Bindl et al., 2019) and resource (Tims et al., 2012) job crafting, this study identifying ten dimensions of ambidextrous job crafting - namely, promotion- and prevention-oriented cognitive, emotional, skill, task, and relational job crafting–our study captures the spectrum of strategies employees use to shape their work while responding to calls to link emotions more closely with proactive behaviors (Peng and Wu, 2021). Moreover, using samples collected from the world’s largest developing country, the current study provides additional support for the dual-orientation structure and confirms the cross-cultural applicability of ambidextrous job crafting (Bindl et al., 2019; Lichtenthaler and Fischbach, 2019).

Second, the Ambidextrous Job Crafting Scale can serve as a diagnostic tool that enables employees to monitor and manage their own behaviors. Employees with prevention-oriented emotional crafting should be able to avoid the constraining effect of negative feelings on their cognition and hence benefit from an increased intention of seeking positive meanings in their future work. As the prevention-oriented job crafting negatively impacts individual work meaning and positively impacts turnover intention, the new scale enables individuals to gain insights into their own proactive behavior and make necessary adjustments to improve it, which in turn can boost their positive work outcomes, such as perceived work meaning. Furthermore, although promotion-oriented job crafting is essential, self-regulated mechanisms are needed to leverage and guide efficacy beliefs in a manner that motivates ambidextrous behavior. Individual ambidexterity is a key employee competence in the contemporary workplace that both individuals and their employers should recognize more explicitly in their efforts to develop work and leadership practices.

Third, this research offers a new angle as well as a scientific measurement tool for managers when implementing ambidextrous job crafting management initiatives. The analyses show that ambidextrous job crafting comprises two distinct forms: promotion- and prevention-oriented, implying that managers should focus on both employees’ promotion- and prevention-oriented job crafting. Managers can use the new ambidextrous job crafting scale to effectively measure, monitor, and manage employees’ proactive behaviors in organizations and conduct interventions promptly. Such efforts might help organizations to avoid negative outcomes, such as emotional exhaustion and turnover intention, and promote more harmonious labor-management relations.

### Limitations and future research recommendations

Like all studies, this research suffers from some limitations. First, in the predictive validity test (Study 2), although we collected data on dimensions like work meaning, emotional exhaustion, and turnover intention from multiple sources at different time points to lessen the same-source bias, the other three criterion variables (work meaning, emotional exhaustion, and turnover intention) were self-rated by employees - a technique that cannot rule out common method bias. Future research may conduct a multi-time, multi-source questionnaire survey to reduce this common method variance.

Second, we did not test the antecedent variables of ambidextrous job crafting in this study. Some antecedent variables might include personal crafting motivation, such as individual needs (Wrzesniewski and Dutton, 2001), and organizational elements, such as superiors’ leadership style (Wang et al., 2017). Future research is recommended to include baseline measurement to eliminate possible bias.

Besides, we examined only three criterion variables, i.e., work meaning, emotional exhaustion, and turnover intention. Future research could extend this work by considering additional outcomes, such as physical health, organizational citizenship behaviors, and organizational performance. Moreover, because emotions are inherently dynamic, researchers should move beyond static descriptions and examine fluctuations in employees’ emotional experiences, as well as their antecedents and consequences. The scale developed in this study may facilitate such efforts by enabling future research to investigate the temporal dynamics of ambidextrous job crafting across different time periods.

## Conclusion

In sum, our study responds to the appeal of exploring how promotion- and prevention-oriented job crafting interact with each other and exploring new measures and approaches applicable to different levels and contexts, and points to new directions in studying individual ambidexterity and addressing effective ways of job crafting.

## Data Availability

The raw data supporting the conclusions of this article will be made available by the authors, without undue reservation.
