# Supplementary material for: Employee ambidextrous job crafting in organizations: theoretical model, scale development, and dual-path effects mechanisms
Source: Front Psychol. 2026 May 1;17:1767375. doi: 10.3389/fpsyg.2026.1767375 (PMC13176212; doi:10.3389/fpsyg.2026.1767375)
Supplement: Supplementary file 1 [file Supplementary_file_1.pdf]

Appendix A. Items Used in the Ambidextrous Job Crafting Scale

**Promotion-oriented cognitive crafting**	**Prevention-oriented cognitive crafting**
Q1 I think about how my work could contribute to our whole society. [d]	Q5 I think working is just a way to make a living.
Q2 I think about new ways of viewing my overall job.[d]	Q6 I focus on the best parts of my job, while trying to ignore those parts I don't like.[d]
Q3 I think about how my job could contribute to my personal growth.	Q7 I think I should limit my role at work.[e]
Q4 I think about how my job could contribute to the organization's goals. [d]	Q8 I try to think of my job as a set of separate tasks, rather than as a "whole."[d]
**Promotion-oriented emotional crafting**	**Prevention-oriented emotional crafting**
Q9 Rediscovering the value of my job would inspire my passion for work.	Q12 When I want to deal with negative emotions, I change my understanding of the situation.[a]
Q10 Viewing the obstacles in my work positively would motivate me to work even harder.	Q13 When I want to deal with negative emotions, I learn to live with it.[h]
Q11 Having coherent career goals and life goals would motivate me to work even harder.	Q14 When I want to deal with negative emotions, I tell myself that they would eventually be gone and that helps me to calm down. [g]
	Q15 When I'm faced with a stressful situation, I turn to other substitute activities.
	Q16 When I feel negative emotions, I make sure not to express them.[a]
**Promotion-oriented skill crafting**	**Prevention-oriented skill crafting**
Q17 I try to actively develop diverse skills in my work.[d]	Q22 I channel my efforts at work towards maintaining a specific area of expertise.[d]
Q18 I try to learn new things at work.[c]	Q23 I make sure that I am capable of fulfilling the duty assigned to me.
Q19 In addition to the core skills required by my job, I try to learn other new things at work. [d]	Q24 I try to keep up with the latest developments in the core discipline related to my job.[d]
Q20 I seek out opportunities for extending my overall skills at work.[d]	Q25 I make sure to be aware of and be capable of coming up with alternative solutions to the same problem.
Q21 I make sure that I use my capacities to the maximum.[c]	Q26 I made sure to have the ability to respond to emergency situations.

**Promotion-oriented task crafting**	**Prevention-oriented task crafting**
Q27 I actively expand the scope of my job responsibilities.[b]	Q31 I actively reduce the scope of my job.[d]
Q28 I spend more time on tasks I enjoy.[b]	Q32 I set aside the difficult tasks for the time being.[f]
Q29 I undertake or seek for additional tasks.[b]	Q33 I actively reduce my off-duty work.
Q30 I actively improve work efficiency.[b]	Q34 I actively reduce the difficulty of the work.[e]
**Promotion-oriented relationship crafting**	**Prevention-oriented relationship crafting**
Q35 I actively seek to meet new people at work.[d]	Q38 I try to avoid situations where I have to meet new people.[d]
Q36 I try to spend more time with a wide variety of people at work.[d]	Q39 I communicate less with people who do not fully support my personal work objectives.[f]
Q37 I actively share resources/messages with others.[b]	Q40 I change my work so that I only interact with people that I feel comfortable working with.[d]
	Q41 My communication with my colleagues is only limited to our jobs.

Note. This final, 41-item based job crafting questionnaire contains two items taken or adapted from Gross et al (2003; noted with superscript a), five items from Berg, Dutton and Wrzesniewski (2013; noted with superscript b), two items from Tims, Bakker and Derks (2012; noted with superscript c), fifteen items from Bindl et al. (2019; noted with superscript d), two items from Laurence (2010; noted with superscript e), two items from Weseler and Niessen (2016; noted with superscript f), one item from Catanzaro (1990; noted with superscript g), and one item from Neubert et al. (2008; noted with superscript h). In addition, these were complemented with 11 newly developed items, based on the definitions of the theorized dimensions, as well as feedback from open-ended survey and experts in the field.
